# A simple economic and heat transfer analysis of the nanoparticles use

**DOI:** 10.1007/s11696-017-0234-4

**Published:** 2017-06-27

**Authors:** Sylwia Wciślik

**Affiliations:** Department of Environmental Engineering, Kielce University of Technology, Aleja Tysiąclecia Państwa Polskiego 7, 25-314 Kielce, Poland

**Keywords:** Nanoadditives, Nanofluid costs, Leidenfrost temperature, Droplet model

## Abstract

In this paper, a review of the impact of most common nanoparticles on the Leidenfrost temperature *T*_*Leid*_ in heat transfer applications is delivered. Moreover, a simple economic analysis of the nanoparticles use is proposed. When coolant is distilled water, *T*_*Leid*_ can range 150–220 °C; occasionally, it can even amount to over 400 °C. When the base liquid is modified by additives, considerable changes in the character of heat transfer are observed. Out of five nanofluids under consideration in this study, the best thermal effect (up to 50%) is obtained when Al_2_O_3_ nanofluid having particle sizes ~39 nm and volume concentration of 0.1% is used. Conversely, the fluid containing TiO_2_ particles, 20–70 nm in size, seems to be the worst of the analysed fluid, giving only 7% heat transfer enhancement in comparison with water. However, when TiO_2_ nanoparticles are far smaller, very good thermal effects are obtained (23–25%). In a majority of the cases analysed, the temperature that marks the onset of film boiling is inversely proportional to concentrations of nanoparticles, which is relevant from the economic standpoint.

## Introduction

Liquids most commonly used in heat transfer systems so far are water and ethylene glycol. However, due to relatively low thermal conductivity, they do not ensure fast and effective heat transfer necessary in modern equipment used in thermal engineering. Depending on the use, different requirements are posed for the equipment. It is usually extremely difficult, or even impossible, to fully meet those requirements. Values of some parameters can be modified by additives of different type. In some instances, change in properties can be achieved by small, pre-determined inclusions. Substances with additives form solutions or suspensions.

To improve heat transfer conditions, different additives to the base liquid are sought. Suspension with particles ranging 1–100 nm (nanoadditives) is termed nanoliquid. For the first time, this term was used by the researches from the Argonne National Laboratory in 1995. Compared with water or suspensions containing particles of the order of mili- or micrometer, nanoliquids show better stability, more advantageous rheological properties and much higher thermal conductivity.

Nanoliquids are used in many industries, everyday applications, and in medicine. Primarily, however, those are considered to be alternative and innovative solutions for thermal and flow devices, in which enhanced transfer of thermal energy is needed. Those include systems of heating and cooling with heat exchangers, solar devices, process chemistry (Ding et al. 2007), and others (Timár et al. 2014).

In recent years, many researchers all over the world have attempted to numerically (Kamyar et al. 2012) and experimentally (Xing et al. 2015) investigate thermophysical properties of nanoliquids (heat transfer coefficient, viscosity, density, specific heat, surface tension, among others) with respect to heat transfer enhancement in thermal engineering devices (Sundar et al. 2013). Wetting angle and wetting conditions also constitute a very important parameter (Cieśliński and Krygier 2013).

One of the recent papers on the subject (Kang et al. 2016) concerns liquid evaporation under film boiling conditions and provides an analysis of distilled water boiling heat transfer on well-wetting surface under atmospheric pressure.

Quenching simulation was intended to determine heat transfer magnitude in film boiling (above the Leidenfrost point), i.e. heat transfer coefficient *α*, minimal value of heat flux density *q*_*Leid*_ and temperature which determine the onset of the second boiling crisis *T*_*Leid*_. Those were determined for three surfaces of the rod, namely hydrophilic, sandpapered to a matte finish, and rough ones. It was found out that the parameters above had higher values for the rod hydrophilic surface than for two other samples having greater roughness. At superheating of ∆*T* = 504 °C, for the matte-finished rod, *q*_*Leid*_ is 158%, *T*_*Leid*_ is 153%, and *α* is 132% higher. The values of those parameters are similar for a rough-surfaced rod.

High-speed imaging used for hydrophilic surface quenching made it possible to observe instability of the process at the liquid–vapour interface. It is necessary to investigate this issue in similar systems in which the Leidenfrost phenomenon occurs, e.g. in quenching.

Stable film boiling can be easily disturbed, which affect results, among others, from surface roughness (*ε*_max_). In many studies on cooling and heating issues, surface roughness effect is disregarded (Nagai and Nishio 1996), whereas some researchers claim this is a significant parameter (Bernardin et al. 1997).

The impact of surface conditions on basic parameters describing liquid film boiling, i.e. *α*, *q*_*Leid*_, *T*_*Leid*_, is also discussed in Kim and Buongiorno (2010). However, the dependences are not correlated, or clearly interpreted.

Taking into account the content of this paper and the author’s field of interest (Orzechowski and Wciślik 2014), the film boiling regime is of key importance. That is related to the evaporation of liquid droplets placed on the surface having the temperature higher than the Leidenfrost point, which for water is *T*_*Leid*_ ~ 220 °C (Wachters et al. 1966). Under actual conditions, however, it is difficult to specify the temperature, because of the lack of clear-cut boundary between boiling ranges. Consequently, the value of temperature that marks the minimum heat flux density in the boiling curve, which corresponds to the onset of stable film boiling, is often not given unambiguously. For instance, study (Hsieh et al. 2016) reported that for distilled water this value was only *T*_*Leid*_ ~ 205 °C.

One of the basic parameters that indicate heat transfer enhancement in technical or technological systems using vapours of nanofluid coolants is the Leidenfrost temperature, which must be correctly determined and interpreted for various nanoparticles concentrations. In processes involving a change of phase, e.g. thermal treatment of steel, inadequately selected model parameters and input data can lead to the deterioration in the properties of the end-product, or even its failure (Wciślik 2014). Additionally, errors in the initial analysis of the properties of nanofluids having different additive concentrations can, under actual conditions, generate high operational costs of installations. In some cases, the presence of nanoparticles does not affect heat transfer conditions.

One of the objectives of this study is to show how the presence of nanoparticles alters the Leidenfrost temperature, thus influencing heat transfer rate and conditions.

The paper also makes an attempt to provide a general, initial analysis of the costs related to the operation of an installation for five most frequently used nanofluids.

## Leidenfrost droplet model

For proper understanding of the nature of the phenomenon, it is essential to give a short review of the basic Leidenfrost droplet models proposed in the literature.

When a liquid droplet evaporates from the surface having the temperature above the second critical point (on a typical boiling curve) MHF (Nemsilová et al. 2014), the amount of vapour produced is sufficient to generate the lift force that supports the droplet. In this case, surface wetting is practically not observed because of a vapour cushion that separates the droplet from the surface. Additionally, surface energy of the droplet is minimised when it assumes a spherical shape.

**Fig. 1:**
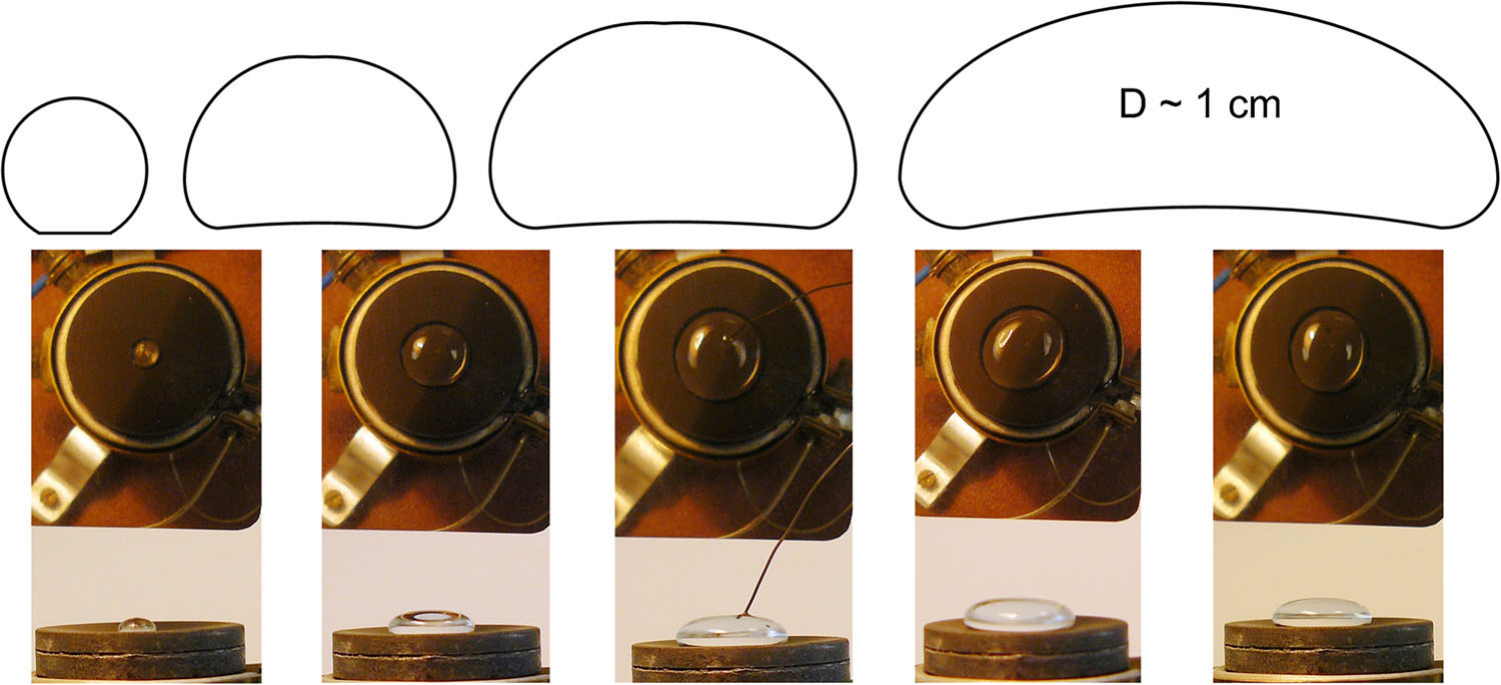
Different forms of Leidenfrost droplet (from *more spherical* to *flat disc*)

The analysis of the geometry of the vapour layer below the Leidenfrost droplet was presented in Burton et al. (2012). The investigations concerned droplets that varied in size and shape (Fig. 1). They were evaporated from the heating surface that had temperature ranging from 245 to 370 °C, at atmospheric pressure.

**Fig. 2:**
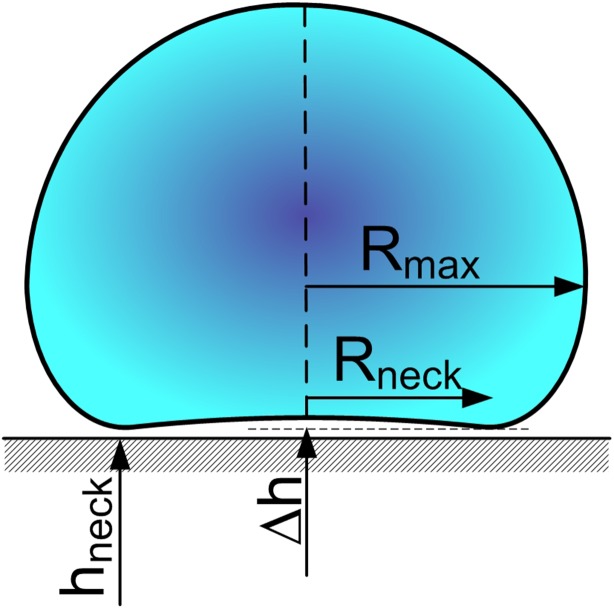
Exemplary Leidenfrost droplet model. notation in accordance with (Burton et al. 2012)

To provide a mathematical description of the phenomenon, in (Burton et al. 2012) a droplet model, shown Fig. 2, was proposed.

The outcome produced by the measurements included, among others, the droplet maximum radius *R*_*max*_, the height of the vapour pocket between the droplet and the surface *h*_*neck*_ (5–100 µm), radius of vapour-induced neck of the droplet *R*_*neck*_, thickness of the vapour pocket ∆*h*.

In the study (Burton et al. 2012), the motions inside the droplet were disregarded, and the results obtained do not refer to larger droplets, for which *R*_*max*_ > ~1 cm.

In the literature, many forms of Leidenfrost droplets can be found. The flattened disc model is often reported (Madejski and Staniszewski 1971). The available models, however, do not account for the presence of nanoadditives which considerably affect the vapour layer thickness, alter the wettability and disturb vapour flow beneath the droplet.

## Leidenfrost temperature conditions in nanofluids

Leidenfrost temperature is defined as the minimal surface temperature necessary to maintain film boiling. Stable film boiling starts at the arrival of liquid droplets onto the hot surface. This boiling regime is characteristic of the stagnation region of liquid droplets impinging on the hot surface, the so-called jets, which can be observed, e.g. in spray quenching.

The correlation proposed by Baumeister and Simon in study (Baumeister and Simon 1973) is considered to be one of the first mathematical dependences (Eq. 1) for determining Leidenfrost temperature:

1 $$ T_{Leid} = T_{l} + \frac{{0.844 \cdot T_{c} \left\{ {1 - \exp \left[ { - 0.016\left[ {\frac{{(\rho_{w} /M_{A} )^{1.33} }}{\gamma }} \right]^{0.5} } \right]} \right\} - T_{l} }}{{\exp (3.066 \cdot 10^{6} \beta )\,erfc\,(1758\sqrt \beta )}} $$

where *T*_*Leid*_ *is* the Leidenfrost temperature, *T*_*l*_ is the temperature of the liquid, *T*_*c*_ is the critical temperature of the liquid, *ρ*_*w*_ is the substrate density, *M*_*A*_ is the atomic mass, *γ* is the surface tension, and *β* = (*λ*_*w*_ *·c*_*w*_ *·ρ*_*w*_) − 1 is the coefficient of solid body properties.

It was agreed that the formula proposed could be applicable, under actual conditions, to liquid metals, superconductors, hydrocarbons and water.

Investigations in Kim and Buongiorno (2010) show that at low values of heat flux density, close to the second critical point, disturbances occurring at phase boundary may produce an instantaneous contact of liquid and the heating surface. Interaction time and its consequences depend on the thermophysical properties of the surface and the presence of nanoparticles. In this case, it is difficult to unambiguously specify the Leidenfrost point temperature. In the literature, different correlations are provided for single-component liquids (Berenson 1961); however, those do not produce real results. That is a consequence of instability in the vapour layer underneath the droplet, caused by the presence of nanoparticles.

Accurate determination of the Leidenfrost temperature for a given liquid seems a very difficult task because of non-linearity of thermodynamic parameters that affect this temperature. In addition, it is necessary to find the extremum of the local heat transfer coefficient or heat flux densities in the boiling curve (Orzechowski and Tyburczyk 2013). The computational algorithm for multicomponent liquids containing nanoparticles is even more unstable. The literature does not provide accurate correlations capable of unambiguously and physically explaining the impact of nanoparticle concentration on *T*_*Leid*_.

Table 1 lists exemplary values of *T*_*Leid*_ for the base liquid and for the liquid with different concentrations of most popular nanoparticles.

**Table 1:** Film boiling incipience temperature, *T*_*Leid*_ for nanofluids with various concentrations of additives

Nanofluid	Mean nanoparticle size, nm	Volume fraction, %	Leidenfrost temperature, *T*_*Leid*_ (LFP), °C			Impact on heat transfer^a^
			(Hsieh et al. 2016)	(Kumar et al. 2015)	(Mitra et al. 2012)	*T*_*Leid*_ increase, %
Pure DI	–	0	204.8	150	407	–
Ag	10–50	0.1	265.1	–		23
		0.07	266.8			23
		0.04	269.3			24
Al_2_O_3_	5–30	0.1	263.2			22
		0.07	267.5			23
		0.04	270.3			24
	38.8	0.1	–	150–300	–	0–50
TiO_2_	10–30	0.1	267.4	–		23
		0.07	270.5			24
		0.04	273.1			25
	20–70	0,1	–	–	437	7
SiO_2_	10–25	0.1	265.8	–		23
		0.07	269.5			24
		0.04	273.1			25
	32.9	0.1	–	150–275	–	0–45
C-diamond	165.4	0.1	–	150–180	–	0–17

^a^Compared with *T*_*Leid*_ for water

One of the important studies on the subject is (Hsieh et al. 2016). The correlation proposed in the study (Eq. 2) makes it possible to specify dimensionless parameters *α* and *q* while taking into account a narrow range of nanoparticle concentrations, which allows the comparison of heat transfer enhancement ratios.

2 $$ \frac{\alpha }{{\alpha_{\hbox{max} } }} = 1.1\,\left( {\frac{q}{{q_{\hbox{max} } }}} \right)^{0.49} \left( {\frac{\phi }{{\phi_{\hbox{max} } }}} \right)^{0.23} $$

where *α* is the heat transfer coefficient, and *q* is the heat flux, *ϕ* nanoparticle volume fraction.

The results (Hsieh et al. 2016) obtained indicate that for distilled water, the use of additives improves cooling performance and enhances heat transfer.

Moreover, dimensionless heat transfer coefficient *α* increased to 1.7, and the heat flux critical density *q*_*max*_ to 1.84 (*q*_*max*_ increase from 227 to 350 W/cm^2^). Those quantities were calculated and given as dimensionless according to Eq. 2, which accounts for nanoparticle volume fraction in the suspension *ϕ* = 0.04–0.1%. As the additive concentration in the base liquid increases, the rate of heat transfer from the surface grows considerably every time. Additionally, film boiling is observed for a relatively short time, which may result from changes in morphology and wettability conditions on the heat transfer surface (Bolukbasi and Ciloglu 2011), and which requires further investigations.

**Fig. 3:**
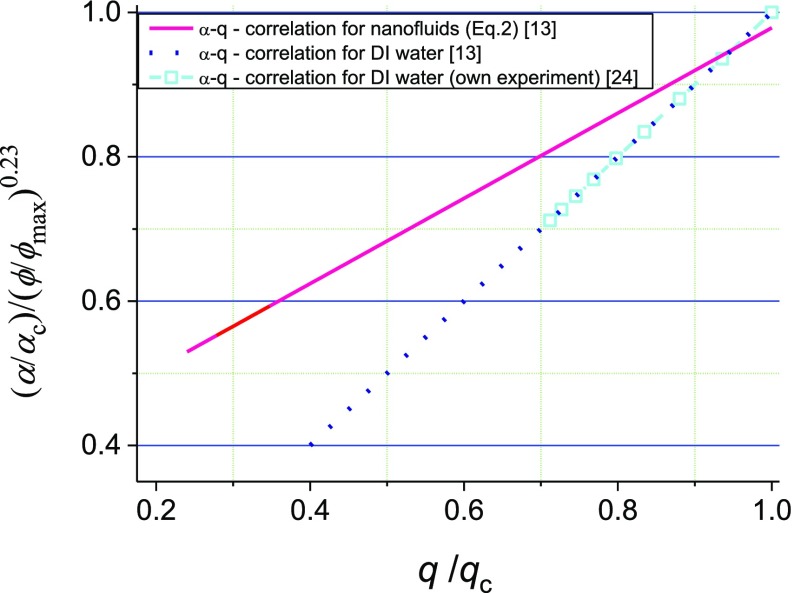
Results of *α* -*q* correlation for water with various nanoadditives concentrations and pure DI water for two cases

It would be logical to check whether the formula above holds under stable film boiling conditions, and how nanoparticle concentration affects heat transfer performance in non-stationary systems. The author’s investigations indicate a very good congruence between the results and the literature data (Hsieh et al. 2016), which can be seen in Fig. 3. The methodology of the determination of non-stationary heat transfer coefficient developed in (Orzechowski and Wciślik 2013) can be applied to multicomponent liquids.

**Fig. 4:**
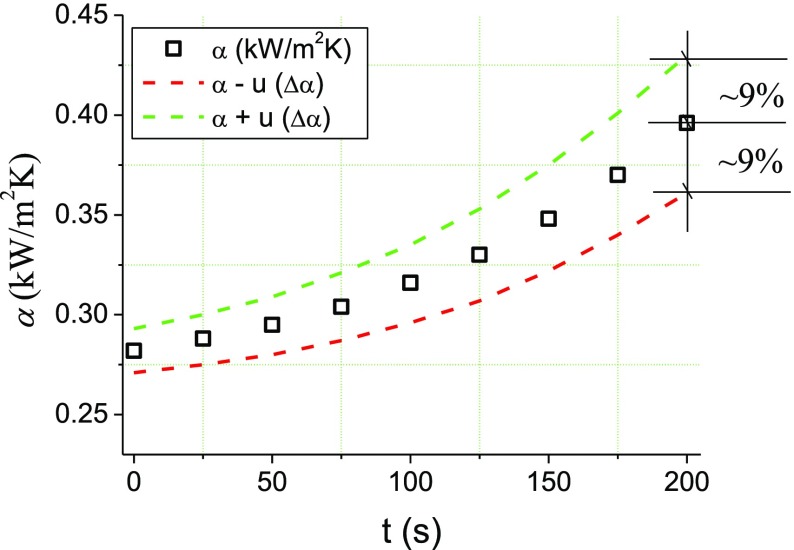
Uncertainty of the heat transfer coefficient measurement for an exemplary series of measurement and copper substrate of *T*_*w*_ = 297.6 °C. on the base of author’s methodology (Hsieh et al. 2016; Orzechowski and Wciślik 2013)

Uncertainties of the measurement of the heating surface temperature and the density of the heat flux generated from the heating surface, determined according to uncertainty propagation law, are ~5%. The overall error in the determination of the heat transfer coefficient *α* is ~13%, which is also confirmed by the author’s investigations and presented in Fig. 4. More detailed calculations can be found in Orzechowski and Wciślik (2014).

## Nanofluids costs—simple calculations

Efficiency of many technological processes can be increased due to the control of the liquid thermophysical parameters by means of nanoadditives, and altered hydrodynamics. Within current state-of-the-art knowledge on nanoliquids, it is not clear what properties nanoliquids have at different concentrations. Those issues need to be thoroughly analysed and the data systematised (Barber et al. 2011). Improperly selected concentrations of additives can generate extra operational costs of the systems. The properties of additives could be then comparable with those of distilled water.

In the paper, an attempt was made to compare the costs of exemplary commonly used nanofluids that vary in additive concentrations. The basic properties of those nanofluids, such as, e.g. density *ρ*, kg/m^3^, necessary to establish the unit price of the solution, were given on the basis of the literature data (Hsieh et al. 2016). It was assumed that the unit price of the nanofluid solution *C*_*Nano*_ depends primarily on the unit cost of the purchase of nano particles *c*_*u*_, EUR/1 g, and unit mass of those particles *m* = *ρ V*. In the proposed formula (3), the term *C*_*other*_ accounts for variable additional costs related to the preparation of the solution. Those costs can change considerably depending on the operational conditions of the system and the means of condition stabilisation. The cheapest and the least energy-consuming systems are those that rely on surfactants (Sarsam et al. 2016). However, the latter are not applicable to high-temperature systems. Then, the use of ultrasonic cleaners and mechanical stirrers generates purchase and operational costs of such devices. In view of the above, the term *C*_*other*_ is disregarded further on in the analysis. The total unit costs *C*_*T*_ of nanofluid preparation are given on the basis of formula (4), which also accounts for the unit purchase price of the base liquid *C*_*Base*_.

3 $$ C_{Nano} = \frac{{c_{u} \rho V}}{0.001} + C_{other} ,\,\frac{EUR}{{dm^{3} }} $$

4 $$ C_{T} = C_{Nano} + C_{Base} ,\,\frac{EUR}{{dm^{3} }} $$

where *C*_*Nano*_ is the unit price of the nanofluid, *c*_*u*_ is the unit cost of the nano particles purchase in EUR/g, *ρ* is the liquid density, *V* is the volume, *C*_*other*_ is the additional costs, *C*_*T*_ is the total unit costs, and *C*_*Base*_ is the base liquid unit price.

It should be added that purchase costs per unit of nanoparticles and also the base liquid (i.e. deionised water) were given on the basis of recently observed market prices. The results of calculations are shown in Table 2. It can also be seen how the price of the preparation of 1 m^3^ of the nanoliquid changes depending on the concentration of nanoparticles of specific size. The choice of nanoparticles was determined by the availability of thermophysical data from the literature. Gross prices include 23% VAT rate.

**Table 2:** Total gross unit costs of the preparation of nanofluids with various nanoparticle concentrations

No.	Nanofluid	Nanoparticle size, nm	Nanofluid gross costs, EUR		Concentration, vol, %	Market net unit price of material, EUR
			of 1 dm^3^, EUR	of 1 m^3^, EUR		
1	Pure DI	–	0.07	70.76	–	54.49
2	TiO_2_	4–8	3.03	3034.21	0.04	10 g ~ 60.47
			5.26	5256.79	0.07	
			7.48	7479.37	0.1	
3	Al_2_O_3_	<50	1.4	1403.74	0.04	10 g ~ 27.21
			2.4	2404.91	0.07	
			3.4	3403.21	0.1	
4	SiO_2_	10–25	1.19	1186.35	0.04	50 g ~ 113.95
			2.02	2024.47	0.07	
			2.86	2862.58	0.1	
5	Ag	<100 nm	223.47	223,473.09	0.04	1 g ~ 455.81
			391.01	391,010.53	0.07	
			558.54	558,542.26	0.1	

## Conclusions

The major objective of the paper was to emphasise the differences in heat transfer performance in the nanofluid systems in which stable film boiling of the coolant occurs. Quenching is an example of such processes. Leidenfrost temperature indicates that the liquid has reached its critical point (film boiling incipience). Leidenfrost temperature varies depending on the installation input parameters, and principally on the liquid properties. The latter can be modified due to different additives, including nanoparticles. Five most frequently used nanofluids having different concentrations and nanoparticle sizes were compared in the study. The results obtained in the investigations were referred to the base liquid, which is most frequently distilled water. The following observations were made:

- both the concentration and size of nanoparticles substantially affect *T*_*Leid.*_
- Al_2_O_3_ nanofluid having volume fraction of 0.1% and particle size of 38.8 nm produces even 50% increase in *T*_*Leid*_ when compared with DI water; similar effects can be found for SiO_2_ with the particle size of 32.9 nm.
- within a specified range of nanoparticle sizes, *T*_*Leid*_ is inversely proportional to the suspension volume fraction, for instance for TiO_2_ and concentrations ranging 0.04–0.1%, it increases by Δ*T*_*Leid*_ = 2%, and with respect to DI water Δ*T*_*Leid*_ = 68.3 K, which gives Δ*T*_*Leid*_ = 25%.

The second part of the paper gives a comparison of costs involved in the preparation of nanofluid. A functional dependence was proposed which includes unit costs of the nanoadditives, the base liquid and other variable and one-off costs that result mainly from stabilisation conditions. Except for nanofluid with Ag particles, the TiO_2_ nanofluid proved the most expensive one; at 0.04% concentration, its unit price, including base fluid, is *C*_*T*_ = 3034.21 EUR/m^3^. However, comparable cooling effect can be obtained using SiO_2_ particles (Table 1), which provides the cheapest solution. For all the options considered, the preparation of SiO_2_-based nanofluids will be ~50% cheaper than TiO_2_ nanofluids.
